# Functional sites for anesthetics in GABA_A_ receptors

**DOI:** 10.18632/oncotarget.14616

**Published:** 2017-01-13

**Authors:** Maria C. Maldifassi, Erwin Sigel

**Affiliations:** Institute of Biochemistry and Molecular Medicine, University of Bern, Bern, Switzerland

**Keywords:** GABA, γ-aminobutyric acid, GABAA receptor, γ-aminobutyric acid type A receptor, anesthetics, Neuroscience

**Figure 1:**
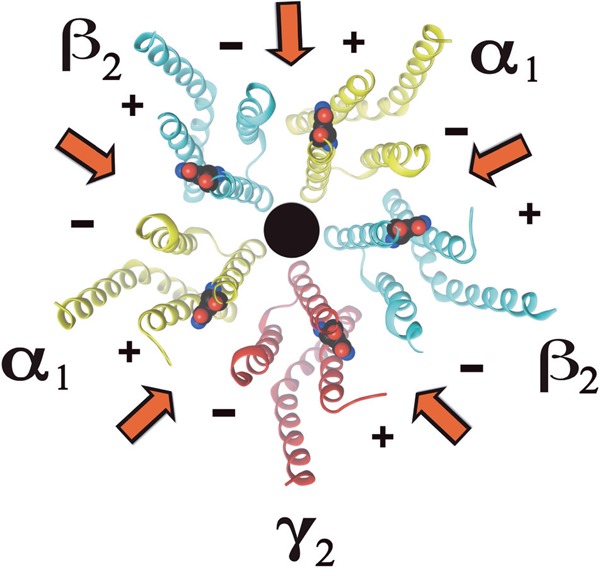
Schematic representation of α1β2γ2 GABAA receptors: Cross section of the receptor with the extracellular domain removed. Shown in blue are residues mutated in each subunit. Arrows indicate the subunit interfaces.

A key target for the intravenous anesthetics propofol and etomidate is the γ-aminobutyric acid type A (GABA_A_) receptor [1]. GABA_A_ receptors are the most important inhibitory neurotransmitter receptors in the central nervous system. They are composed of five subunits that surround a central Cl^-^ selective ion channel. Each subunit has an extracellular domain, four trans-membrane domains, and a variable-size intracellular loop. The major receptor isoform is composed of α_1_, β_2_, and γ_2_ subunits, arranged counter clockwise γ_2_β_2_α_1_β_2_α_1_ as seen from the cell exterior [for review see 2 and references therein]. In α_1_β_2_γ_2_ GABA_A_ receptors there are five subunit interfaces: two β+/α- interfaces, and one of each α+/β-_,_ α+/γ-, and γ+/β-; where the sidedness of the subunits is designated + and -. Figure 1 shows a schematic representation of a cross section of the receptor near the extracellular surface of the membrane. Two GABA binding sites are located at the β+/α- extracellular subunit interfaces.

Earlier efforts to identify binding sites for intravenous anesthetics on GABA_A_ receptors used mutational analysis, sometimes combined with cysteine modification, or photoaffinity labeling [for review see 3 and references therein]. Photoaffinity labeling using photo-reactive analogs of anesthetics is a powerful method to identify amino acid residues located in or close to the binding pocket of the anesthetic by an irreversible covalent reaction. All residues identified with this method were located at subunit interfaces in the trans-membrane domain. Although this method has the advantage that it is able to point out single amino acid residues involved in binding, it largely ignores their functional relevance.

Until now mutational approaches have concentrated on a limited number of subunit interfaces, without resolving them. One of the residues identified here was β_2_N265. We used mutation of this and homologous residues in other subunits as reporter mutations to investigate the functional importance of all subunit interfaces for potentiation by the anesthetics etomidate and propofol [4]. The mutations were N265I, in the β_2_ subunit and S269I and S280I in the α_1_ and γ_2_ subunits. Receptors were expressed in *Xenopus* oocytes, and characterized using two electrode voltage-clamp electrophysiology.

In the triply mutated receptor, which combines mutations at all interfaces, potentiation by both anesthetics was eliminated [4]. In receptors carrying the reporter mutation in the α+/β- or α+/γ- interfaces, i.e. α_1_S269Iβ_2_γ_2_ receptors, potentiation by propofol and etomidate was unaltered [4]. The γ_2_S280I mutation, which reports on the involvement of the γ+/β- interface, altered the potentiation of both anesthetics [4], thus indicating that these anesthetics are acting at this interface. Photoaffinity labeling propofol analogs has also identified residues in the γ+/β- interface [3, 5], but the importance for function was not clear.

Introduction of the β_2_N265I reporter mutation located at both β+/α- interfaces, eliminated potentiation by etomidate and propofol [4]. Several point mutations of β_2_N265 have shown its importance of this residue for modulation by etomidate and propofol [see 3, 4 and references therein], though no photoaffinity labeling has ever been observed. Labeling of other residues at the β+/α- interface(s) has indicated presence of binding sites for etomidate and propofol [3, 5], while the contribution of individual sites could not be differentiated. By using receptor concatenation and the β_2_N265I mutation, we individually altered one of the two sites, and were able to dissect the functional contribution of the two β+/α- interfaces. Etomidate acted almost exclusively at the β+/α- interface flanked by γ and β subunits with a minor contribution of the β+/α- interface flanked by α and γ subunits [4]. In contrast to etomidate, both interfaces were similarly important for modulation by propofol.

The importance of the corresponding binding pockets for anesthetic action is underlined by the fact that recently two new allosteric modulators of GABA_A_ receptors were identified that excert their action exclusively through the β+/α- interfaces. Similar to etomidate they predominantly act at the β+/α- interface flanked by γ and β subunits, but in contrast to etomidate they do not act at the γ+/β- subunit interface. Both substances induced loss of righting reflex in *Xenopus laevis* tadpoles with potencies similar to propofol [6] and represent leads for the development of novel anesthetics.

In summary, we have demonstrated that etomidate exerts its function predominantly through the γβ+/α-β and γ+/β- subunit interfaces while propofol acts predominantly at the γβ+/α-β, αβ+/α-γ and γ+/β- subunit interfaces. GABA_A_ receptors harbor modulatory sites for these anesthetics in three of the five-subunit interfaces. The anesthetics etomidate and propofol use a different sub-set each. Pentobarbital seems to use at least one additional interface [4]. Taken together, these data demonstrate the existence of an asymmetry in potentiation of the α_1_β_2_γ_2_ GABA_A_ receptor by these popular intravenous anesthetics.
